# Development and validation of a French questionnaire concerning patients’ perspectives of the quality of palliative care: the QUALI-PALLI-Patient

**DOI:** 10.1186/s12904-019-0403-z

**Published:** 2019-02-11

**Authors:** Frédéric Guirimand, Patricia Martel-Samb, Christian Guy-Coichard, Stéphane Picard, Bernard Devalois, Laure Copel, Anne Abel, Véronique Ghadi, QUALI-PALLI group and Philippe Aegerter

**Affiliations:** 1 Pôle recherche SPES “Soins Palliatifs en Société”, Maison Médicale Jeanne Garnier, 106 avenue Emile Zola, 75015 Paris, France; 2 Unité de Recherche Clinique URC HU PIFO, AP-HP, Hôpital Ambroise Paré, Boulogne, France; 3 Équipe Mobile de Soins Palliatifs, AH-HP, Hôpital Saint Antoine, Paris, France; 4 Unité de Soins Palliatifs, Groupe Hospitalier Diaconesses Croix Saint-Simon, Paris, France; 5 Centre de Recherche Interprofessionnel Bientraitance et Fin de Vie, Service de Médecine Palliative, Hôpital de Pontoise, Pontoise, France; 6 Équipe Mobile de Soins Palliatifs, AP-HP, Hôpital Ambroise Paré, Boulogne, France; 7 Haute Autorité de Santé, Saint Denis, France; 8 GIRCI-IDF, Cellule Méthodologie, Paris, France; 9 UVSQ, UMR-S 1168 Université de Versailles Saint Quentin Paris Saclay, INSERM VIMA Aging and Chronic Diseases, Epidemiological and Public Health Approaches, Montigny-le Bretonneux, France

**Keywords:** Palliative care, Questionnaire, Quality of care, Satisfaction, Validation, Outcome assessments, Patient reported outcome measures

## Abstract

**Background:**

Indicators for the quality of palliative care are a priority of caregivers and managers to allow improvement of various care settings and their comparison. The involvement of patients and families is of paramount, although this is rarely achieved in practice. No validated assessment tools are available in French. Simple cultural adaption of existing questionnaires may be insufficient, due to the varying organization of care in different countries.

The purpose of this study was to develop and validate a new instrument to measure the quality of palliative care and satisfaction from the patient point of view.

**Methods:**

Results from a qualitative study were used by a multi-professional workgroup to construct an initial set of 42 items exploring six domains. A cross-sectional survey was conducted in seven hospitals, encompassing three care settings: two palliative care units, one palliative care hospital, and four standard medical units with a mobile palliative care team. All items were assessed for acceptability. We conducted exploratory structural analysis using Principal Component Analysis (PCA), and evaluated external validity by comparison against global rating of satisfaction and the MD Anderson Symptom Inventory (MDASI) questionnaire.

**Results:**

A total of 214 patients completed the questionnaire. After removing 7 items from the response distribution, PCA identified eight interpretable domains from the 35 final items: availability of caregivers, serenity, quality of information, pain management, caregivers’ listening skills, psychosocial and spiritual aspects, possibility to refuse (care or volunteers), and respect for the patient. Internal consistency was good or acceptable for all subscales (Cronbach’s α 0.5–0.84), except the last one (0.15). Factorial structure was found globally maintained across subgroups defined by age, sex, Palliative Performance Scale (PPS ≥ 60%, 40–50% and ≤ 30%), and care settings. General satisfaction was inversely correlated with the 2 scores of the MDASI questionnaire: symptoms’ severity and impact on life. Each subscale, except “possibility to refuse”, correlated with general satisfaction.

**Conclusions:**

Quali-Palli-Pat appears to be a valid, reliable, and well-accepted French tool to explore the quality of care and the satisfaction of palliative care patients. It should be confirmed in a wider sample of care settings.

**Trial registration:**

clinicaltrials.gov NCT02814682, registration date 28.6.2016.

**Electronic supplementary material:**

The online version of this article (10.1186/s12904-019-0403-z) contains supplementary material, which is available to authorized users.

## Background

Improving palliative care requires an assessment of both patient needs and the quality of care they receive [1–7]. Although many tools focus on quality of life, notably on physical functioning or psychological symptoms as shown in a systematic review [1], fewer tools explore patient appraisal of their care [8–10]. Patients’ point of view is a major aspect of quality of care, [6, 11–13]. The variety of domains which should be considered by such an outcome measure has been explored (for example: physical, psychological, social, spiritual, cultural, ethical and legal aspects of care) [14, 15] and several tools assess the satisfaction or quality of palliative care, but they may be inadequate to explore the most distressing concerns of patients [16]. Moreover, none are available in French or tailored to French culture [16–21].

The organization of care (legislation, regulation, financing…) and the skills of caregivers (prescribing privileges for advanced practice nurses, for instance) vary between countries [22]. Simple cultural adaptation of existing assessment tools may be insufficient, thus requiring the development of tailored instruments. Quali-Palli is a research project aimed to develop multidimensional quality indicators of palliative care practices [23]. Instead of using predefined attributes determined elsewhere, we used a generative approach to obtain a thorough understanding of what is important concerning quality of care from three groups of stakeholders: patients, families, and healthcare professionals. We previously used a theoretical approach with a qualitative analysis method to develop a quality of care model from the patients’ perspective [23]. This approach highlighted the major dimensions of quality of palliative care for all stakeholders: comprehensive support for the patients themselves, clinical management, involvement of families, and care for the imminently dying person, as well as views of patients, families, and healthcare professionals on death [23]. The aim of the present study is the next step of the Quali-Palli project: to develop and validate a patient questionnaire (Quali-Palli-Pat) to measure the quality of palliative care and satisfaction, based on the assumption that patients are the experts of their own life. It had to be usable in all care settings where palliative patients are hospitalized, since the most of palliative care is provided in a hospital facility [24]. This questionnaire could complement the key elements concerning the quality of palliative care of all stakeholders.

## Methods

### Construction of the questionnaire

For the Qualli-Palli research project, a multi-professional workgroup (physicians, nurses, psychologists, social workers, and volunteers) conducted a literature review to identify research, indicators, and instruments that measure quality of life and quality of care for patients receiving palliative care. We selected a first set of 42 items exploring six domains, based on the major themes that emerged from the palliative literature, as well as from general purpose quality of care questionnaires, and from content analysis of our qualitative study [23, 25]. The six domains consisted of: the quality of information about health status and care (three items), availability of physicians and caregivers (10 items), attitudes of caregivers (six items), physical well-being and symptom management (10 items), functioning of the unit, including the handling of existential issues (nine items), and information and reception of relatives (four items) (Table 1). A four-point Likert scale was displayed after each question with the following ordered responses: no, not at all; not really; almost; yes, exactly, as well as a “does not apply to me” response (not applicable). The last (43rd) item assessed global satisfaction, according to four categories: not satisfied at all; partially satisfied; satisfied; very satisfied. This was followed by an open-ended question about any unmentioned aspects that seemed important to the patient.

**Table 1:** Quali-Palli questionnaire tested by the patients

Information about my health and care	
I have received very clear information concerning:	
Q1	-the evolution of my state of health
Q2	-the objective of the treatments (medication, methods)
Q3	-possible adverse effects of treatments
Relations with the doctors and caregivers	
Q4	I get information easily from everyone on the staff
Q5	The doctors answer all my questions
Q6	I am involved in my own care and decisions concerning me
Q7	I can refuse certain treatments
Q8	The doctors listen to me and consider what I say
Q9	I know the doctor(s) who deal with me
Q10	I see a caregiver as often as I wish
Q11	I see a caregiver as often as I want or need
Q12	The caregivers respond quickly when I call
Q13	They do the maximum when I am anxious, worried, or sad
Attitude of the caregivers	
Q14	They respect my intimacy
Q15	They do everything possible to help me with my daily activities
Q16	I was disturbed by the comments made by the doctors during the visit
Q17	The caregivers are available to listen to me
Q18	The caregivers show genuine availability and attentiveness
Q19	The caregivers sometimes talk to each other as if I was not there
Physical wellbeing	
Q20	I am regularly asked about my pain
Q21	My pain is quickly taken care of when I report it
Q22	If necessary, my pain is taken into account before any nursing care
Q23	The caregivers are gentle when providing care
Q24	I receive appropriate help when I eat
Q25	I receive appropriate help to wash myself
Q26	A bedpan or help to go to the toilet is offered in a respectful way
Q27	The room and the unit are calm and restful
Q28	I can rest as much as I wish
Q29	The caregivers respect my need for rest
Functioning of the unit	
Q30	The nurses do everything they can to make themselves available
Q31	The doctors do everything they can to make themselves available
Q32	I have the impression that the unit is well-run
Q33	The atmosphere in the unit is good
Q34	I can take advantage of the presence of volunteers
Q35	I was able to refuse the presence of volunteers
Q36	I can see a psychologist when I need to
Q37	I can see a social worker when I need to
Q38	I can talk to someone about philosophical or religious issues if I wish
Information/reception of relatives	
Q39	The doctors ask me for my permission before informing my relatives about my health
Q40	My relatives get clear and understandable information about my health
Q41	My relatives are always received well
Q42	The visiting rules suit me
Global satisfaction	
Q43	Overall, what is your level of satisfaction with your care

### Pilot study for face validity

The questionnaire needed to be brief, understandable, and easy to complete for hospitalized palliative-care patients and was designed to be self-administered or completed with informal assistance. This questionnaire was presented to 12 patients from three different hospitals to verify comprehensibility and acceptability of the items and response patterns. The patients were asked to complete the first version and to rate each item for clarity in wording, understanding, and relevance to the quality of care [26].

### Process of the clinical study

A cross-sectional study was conducted in the Paris metropolitan region in seven settings, encompassing three different care structures: two hospital-based palliative care units (Centre Hospitalier de Pontoise; Groupe Hospitalier Diaconesses Croix Saint-Simon, Paris), one palliative care hospital (Maison Médicale Jeanne Garnier; Paris), and four other standard medical units with mobile palliative care (Institut Curie, Paris, Hôpital Ambroise Paré, Boulogne, Hôpital Saint Antoine, Paris, Hôpital Saint Joseph, Paris). Eligible patients were consecutively recruited according to the following criteria: older than 18 years, hospitalized for palliative care for more than 72 h, a life expectancy under 3 months, and able to understand and communicate well in French and to respond to questionnaires.

One of two trained research assistants at each participating site communicated with the staff to identify potentially eligible patients and excluded those who were considered to be cognitively impaired. After assessing eligibility, the research assistant approached the patients, provided written and oral information about the study, invited them to participate, reassured them about confidentiality concerns and the independence of the study from the caregivers and the hospital, and received their consent. The research assistant gave the questionnaire to the patients informing them that they could take as much time as they needed to complete it or helped them to complete it if they were too tired. For validation purposes, patients completed the French version of the MDASI, which is a reliable assessment tool containing 13 items for the assessment of symptoms and six items to measure their global impact on their daily living and quality of life [27]. The completed questionnaires were directly transmitted to the coordinating investigator or addressed to him in a sealed envelope, without any interaction or supervision by professionals of the structure. Demographic (age, sex) and medical (pathology, stage) data were obtained from the caregivers who also informed us of changes in the health status of the patients (worsened, stable, improved) at the retest visit, relative to the beginning. The functional status of the patients was assessed using the French version 2 of the Palliative Performance Status (PPS) [28, 29].

### Statistical analysis

#### Scoring

Data were re-scaled to range between 1 (no, not at all) and 4 (yes, exactly), as we considered that these items are discrete measurements about an underlying continuum [30]. The item scores were inverted for three negatively worded items so that higher scores indicated higher quality. Not applicable (NA) responses and missing data (MD) were regrouped as non-response (NR).

Acceptability was assessed by computing the proportion of missing responses per item, per subscale, and overall, as well as by measuring the time taken to complete the questionnaire.

#### Item selection

The first selection of items was made from the descriptive response distribution for each item. The criteria used to guide item selection/deletion were rates of missing data and ‘not applicable’ responses, ceiling and floor effects, and redundancy. Items for which the missing data rate was < 20% were estimated by multiple imputation based on four covariates: age, sex, structure, and Palliative Performance Scale (R software; package Mice - Multivariate Imputation by Chained Equations) [31, 32].

The impact of the following factors on the descriptive response distribution was studied: age, sex, and PPS score according to three categories (≤30, 40–50, ≥ 60) and the type of structure (palliative care structure (unit or hospital) or standard hospitalization). “Not applicable” responses were sure to be present, due to the any-structure purpose of the questionnaire, so an item that may have been discarded on the global analysis was retained if it showed good properties for one care structure.

The criteria used for removing items from the questionnaire were: (1) a high rate of NR (NA or MD) (≥ 20%), (2) a floor or a ceiling effect defined when a category had more than 90% of the same response; (3) and potential redundancy between items defined by a high Pearson correlation between two items (r ≥ 0.70). Clinical relevance considerations also tempered the selection: interest of the item in the literature, room for improvement or a special focus observed during the qualitative study.

#### Construct validity

The underlying structure among the remaining items was explored using principal component analysis (PCA). Before PCA, the suitability of the data for factor analysis was assessed using Bartlett’s sphericity test, which tests the overall significant differences in the correlation matrix, and the Kaiser–Meyer–Olkin (KMO) measure of sampling adequacy. An orthogonal varimax rotation on the correlation matrix was performed to explore the underlying construct because our purpose was to generate factor scores and we expected weak correlations between factors. The cut-off to determine the number of components resulted from a Cattell’s scree plot, completed by Horn’s parallel analysis using R paran package [33, 34]. Items with a low weight (< 0.35) were removed. The criteria to attribute each item to one factor resulted from predetermined rules: a substantial loading (> 0.35) on one principal component; if an item loaded across several factors, it was attributed to the factor for which it maximized the internal consistency, estimated by Cronbach’s α coefficient; at last, clinical judgment and substantive knowledge were used to make sense.

Indeed, internal consistency was estimated using Cronbach’s α for Quali-Palli-Pat total scores and any identified subscales. An α coefficient between 0.7 and 0.9 was considered to indicate good internal consistency without redundancy of items. Accordingly, we calculated the average inter-item correlation within subscales that should fall within the range of 0.25 to 0.50 and cluster narrowly around the mean value [35]. We also calculated the correlation of items in one subscale with the other subscale scores, considering that they should be lower than within their own subscale. An analysis of the inter-subscale correlation matrix was conducted to confirm independence of dimensions if the coefficient was under 0.40 [36] .

The stability of these factors was assessed by principal-component analysis i) using oblique rotations and ii) within different sub-groups of patients defined by sex, age, PPS (PPS ≥ 60%, 40–50% and ≤ 30%) or care settings. We compared factorial structures across related sub-groups (for instance, men and women) by summing how many items were attributed identically to a given factor between the two related structures. To take into account what may be due to chance, we performed a permutation test by comparing this observed statistic to the distribution of the same statistic across 10,000 sub-groups defined by a random equiprobable binomial variable. The reasoning is that if the null hypothesis of there being no influence of a given characteristic on the factorial structure is true, then permuting the values of this characteristic among individuals produces random noise and should be equally likely to produce a larger or a smaller proportion of identically attributed items; on the contrary if the characteristic has a strong influence, then the factorial structures should be different in the related sub-groups, giving a low proportion of identically attributed items. The significance of the observed proportion of identically attributed items for a characteristic is the proportion of such random permutations that lead to a lower proportion of identically attributed items.

#### Content validity

Experts in the field, i.e. six palliative care physicians, interpreted the results of the PCA, focusing on the clinical sense of the various components, henceforth called subscales. These subscales should reflect domains that are important to patients and other stakeholders and each domain should be represented in at least one subscale. For each subscale, a score was then generated by summing all responses.

#### Reproducibility

Reproducibility was assessed by test-retest procedures that should reflect the stability of the scores over time when no change has occurred in the patient. The time between two tests must be short enough to ensure that no change in the quality of care has occurred and long enough to prevent recall bias. A time of 1 week is generally considered to be appropriate, but feasibility may be a concern in the palliative context, as patients and their needs at the end of life are rarely stable [1].

#### External validity

There is currently no other French-validated or French-translated instrument for measuring quality of palliative care from the point of view of patients. Convergent/divergent validity was measured by comparing our instrument with another instrument that measures a theoretically related, but different, construct. We used i) the responses to the last question, which is a global assessment of satisfaction with care, and mainly ii) an assessment tool of symptoms and their impact, i.e. the French version of the MDASI [27]. However, although symptoms and satisfaction with care are related, no correlation could be hypothesized a priori: the aggravation of symptoms may precede the evolution of satisfaction related to care rather than occurring concurrently [37]. Spearman’s correlation between Quali-Palli-Pat global satisfaction and MDASI symptom scores and impact scores were calculated [38]. Specifically, we verified Spearman’s correlations between Quali-Palli-Pat availability of caregivers and MDASI symptom scores.

### Results

#### Patient characteristics

The 43-item questionnaire was administered to 214 patients, from March 2012 to June 2013. Clinical and demographic details are provided in Table 2.

**Table 2:** Patient Characteristics

Characteristic	Patients (*n* = 214)
Age, years	
mean ± sd	65.9 ± 14.8
Min-Max	18–92
< 55	22%
55–64	18%
65–74	27%
> = 75	33%
Percent men	42%
Setting	
USP	43%
Non-USP	57%
Length of stay in the hospital	
Mean ± std	19.4 ± 21.5
Median [IQ]	13 [6–23]
Length of stay in the service	
Mean ± std	14.1 ± 15.4
Median [IQ]	9 [5–17]
Diagnosis	
Non malignacy	2%
Malignacy	98%
Site of primary cancer	209
Breast	19%
Lung/pleura	18%
other digestive cancer	17%
Colorectal	13%
Gynecological	9%
Hematological	7%
Urogenital	6%
Skin	4%
Other/unknown	2%
ENT	2%
Esophagus	2%
Brain	1%
PPS	
≥ 60%	23%
40–50%	45%
≤ 30%	31%

PPS: Palliative Performance Scale (French version 2)

#### Acceptability and delivery of the questionnaire

The 12 patients of the pilot study completed the questionnaire which was fully understood without difficulty in understanding the vocabulary or meaning of the questions. So the initial version was not modified.

Only 20% of the 214 patients completed the questionnaire themselves: the lower the PPS score, the more patients needed help to complete the questionnaire; only 6% of patients with a PPS ≤ 30 could do it on their own. One hundred and eighty patients (84.1%) provided complete questionnaires without missing data (MD) but most of them gave not applicable responses (NA); only 15 patients completed the entire questionnaire with no MD or NA. The remaining 34 patients did not complete from 1 to 30 items. Globally, 9.3% of the data was considered to be missing (860/9202); the median number of NR was three items (interquartile range IQR 2–5). Eight patients who completed less than 30% of the questionnaire and 14 who answered NR to the entire questionnaire were excluded. The final analysis included 192 patients. The time to fully complete the two questionnaires (Quali-Palli-Pat, MDASI) was between 20 and 25 min.

#### Item selection

Six items had a missing rate > 20%. Nine items obtained the highest level on the Likert scale with a ceiling effect of more than 90% of the responses. According to the rules for removing items, 12 (14, 22, 24, 25, 26, 33, 34, 35, 36, 38, 41, 42) should have been deleted. However, our qualitative study highlighted the importance of items 22 (“If necessary, my pain is taken into account before any nursing care”) and 35 (« I was able to refuse the presence of volunteers »), so we decided to retain both items [23]. Removing all items (24 to 26) related to dependent patients was considered unacceptable; item 24 and 25 were retained, but not 26, which combined two removal rules. Items 36 (” I can see a psychologist when I need to “») and 38 (“I can talk to someone about philosophical or religious issues if I wish”) were also retained because of the clinical importance of psychological or spiritual suffering.

#### Inter item correlations

According to the between items correlation matrix, items 24 (“I receive appropriate help when I eat”) and 25 (“I receive appropriate help to wash myself”) were highly correlated (r = 0.73; *p* < 0.0001); item 24, which had the higher NA rate (59%) was eliminated.

After removing seven items (14, 24, 26, 33, 34, 41, and 42), the questionnaire contained 35 items which were submitted to factorial analysis. Global KMO measure was .78 while items’ KMO were generally good (only one item <.4, median = .76).

#### Construct validity

After the imputation of missing data, PCA identified an eight factor-solution which accounted for 76% of the total variance, according to parallel analysis. One factor (#7) had only two items but with high loadings (> = .65). The matrix of factor loadings is shown in Table 3.

**Table 3:** Principal component factor analysis (varimax rotation) computed using the final 35-item version of the questionnaire

		Factor1	Factor2	Factor3	Factor4	Factor5	Factor6	Factor7	Factor8
Q11	I see a caregiver as often as I want or need	**0.38**	0.08	0.25	0.30	0.31	0.07	−0.21	− 0.11
Q12	The caregivers respond quickly when I call	**0.38**	0.30	0.08	0.29	0.23	0.10	−0.24	− 0.31
Q13	They do the maximum when I am anxious, worried, or sad	**0.48**	0.11	0.13	0.26	***0.35***	0.12	0.24	−0.12
Q15	They do everything possible to help me with my daily activities	**0.58**	***0.36***	0.10	0.25	0.09	0.05	0.25	−0.15
Q17	The caregivers are available to listen to me	**0.61**	0.13	0.10	0.29	0.03	−0.03	− 0.22	0.04
Q18	The caregivers show genuine availability and attentiveness each time they enter the room	**0.65**	0.32	0.12	0.33	−0.06	− 0.02	− 0.01	0.07
Q25	I receive appropriate help to wash myself	**0.56**	0.22	−0.07	−0.16	− 0.05	0.23	0.09	−0.09
Q30	The nurses do everything they can to make themselves available	**0.66**	−0.07	0.11	0.15	0.13	−0.05	− 0.04	− 0.01
Q32	I have the impression that the unit is well-run	**0.54**	***0.37***	0.01	0.09	0.17	−0.07	− 0.07	0.21
Q23	The caregivers are gentle when providing care	0.25	**0.61**	0.16	0.20	0.00	0.10	0.14	−0.13
Q27	The room and the unit are calm and restful	0.15	**0.71**	−0.01	0.14	−0.02	−0.21	− 0.11	0.20
Q28	I can rest as much as I wish	0.12	**0.78**	0.06	0.06	0.12	0.08	−0.05	0.02
Q29	The caregivers respect my need for rest, for example the time they wake me in the morning	0.18	**0.59**	−0.12	0.04	0.18	−0.08	−0.25	− 0.08
	I have received very clear information concerning:								
Q1	- the evolution of my state of health	0.01	0.07	**0.68**	0.07	***0.35***	−0.03	−0.04	−0.04
Q2	- the objective of the treatments (medication, methods)	0.06	0.05	**0.68**	0.23	−0.03	−0.04	0.07	0.03
Q3	- possible adverse effects of treatments	0.15	−0.08	**0.62**	0.06	−0.14	−0.16	0.08	0.01
Q6	I am involved in my own care and decisions concerning me	0.01	0.07	**0.58**	0.08	0.08	0.13	−0.10	0.13
Q20	I am regularly asked about my pain	0.12	0.07	0.19	**0.61**	0.12	0.00	0.20	0.12
Q21	My pain is quickly taken care of when I report it	0.20	0.15	0.03	**0.67**	0.02	0.04	−0.12	0.07
Q22	If necessary, my pain is taken into account before any nursing care	0.16	0.11	0.13	**0.63**	0.03	0.04	0.01	−0.03
Q4	I get information easily from everyone on the staff	***0.39***	0.12	***0.38***	−0.17	**0.41**	0.04	−0.19	−0.07
Q5	The doctors answer all my questions	0.31	0.10	***0.39***	−0.17	**0.47**	−0.05	0.03	0.01
Q31	The doctors do everything they can to make themselves available to me	***0.44***	0.16	0.13	0.13	**0.57**	−0.05	−0.06	0.18
Q39	The doctors ask me for my permission before informing my relatives about my health	0.08	−0.06	0.00	***0.36***	**0.35**	***−0.35***	0.06	0.08
Q40	My relatives get clear and understandable information about my health	0.00	0.09	0.04	0.26	**0.60**	−0.07	0.10	0.08
Q16	I was disturbed by the comments made by the doctors during the visit	−0.05	***0.42***	0.17	−0.11	0.30	**0.48**	0.16	−0.03
Q36	I can see a psychologist when I need to	0.12	−0.01	0.03	0.13	−0.08	**0.66**	−0.01	−0.25
Q37	I can see a social worker when I need to	0.01	−0.20	−0.08	0.08	−0.06	**0.56**	−0.12	0.24
Q38	I can talk to someone about philosophical or religious issues if I wish	−0.02	0.12	−0.25	− 0.19	***0.43***	**0.43**	−0.03	0.11
Q7	I can refuse certain treatments	0.01	−0.07	0.06	0.05	0.08	−0.04	**0.65**	−0.10
Q35	I can refuse the presence of volunteers	− 0.07	− 0.05	− 0.06	−0.02	− 0.03	−0.04	**0.69**	0.21
Q8	The doctors listen to me and consider what I say	0.33	0.04	***0.37***	−0.25	− 0.01	0.02	0.01	**0.44**
Q9	Some care or treatment is performed without my consent	0.21	0.02	−0.06	− 0.10	− 0.15	0.14	− 0.05	**−0.51**
Q10	I know the doctor(s) who take(s) care of me in the service	0.04	−0.06	***0.36***	0.11	0.17	0.06	0.16	**0.39**
Q19	The caregivers sometimes talk to each other as if I was not there	0.33	0.32	−0.04	0.16	− 0.12	0.25	− 0.05	**0.49**

Factor 1: 9 items concerning the availability of caregivers to meet the needs of the patient

Factor 2: 4 items concerning attention to patient serenity: gentleness, rest, environment, and respect for their rhythms

Factor 3: 4 items concerning the quality of information given to the patient (health, care, treatment) and the patient’s involvement in decisions

Factor 4: 3 items related to pain management

Factor 5: 5 items concerning the willingness of caregivers, particularly doctors, to listen

Factor 6: 4 items concerning the psychological, social, and spiritual needs of the patient and, *above all, their need to be respected*

Factor 7: 2 items concerning the possibility of refusing certain care or refusing the presence of volunteers

Factor 8: 4 items concerning the respect of the patient as an actor in their own care

The criteria to attribute an item to one factor resulted from a substantial loading (> 0.35 in boldface) on one principal component. When an item loaded across several factors, it was attributed to the factor for which it maximized the internal consistency; the others loadings are italic

#### Consistency

Table 4 shows the mean score for each factor with a high level of satisfaction (3.4 to 3.8), the normalized Cronbach’s alpha, and inter-item correlation. Internal consistency was very good for factors 1, 2, and 4, acceptable for factors 3, 5, 6, and 7, and low for factor 8. The inter-item correlations within factors were generally close to 0.5 within a narrow band, except for factor 6, and mainly 8 (Table 5). Between-subscale inter-item correlations were consistently lower, except for items 4, 16, 21, and 23. All inter-subscale correlations were lower than 0.4, except those between factors 1 (availability of caregivers) and 2 (serenity) and factors 4 (pain) and 5 (attention to the patient), which were close to 0.5 (Table 6).

**Table 4:** Validity of convergence and reliability

Factor	N items	mean (s.d.)	α Cronbach	Validity of convergent
Factor 1	9	3.7 (0.4)	0.84	0.56 (0.47–0.69)
Factor 2	4	3.5 (0.6)	0.74	0.52 (0.43–0.60)
Factor 3	4	3.4 (0.7)	0.66	0.44 (0.38–0.52)
Factor 4	3	3.7 (0.6)	0.71	0.51 (0.47–0.58)
Factor 5	5	3.5 (0.6)	0.65	0.39 (0.26–0.57)
Factor 6	4	3.8 (0.4)	0.5	0.26 (0.19–0.32)
Factor 7	2	3.8 (0.6)	0.57	0.39
Factor 8	4	3.7 (0.4)	0.15	0.05 (≤0.25)

**Table 5:** Validity of divergence

	Factor 1	Factor 2	Factor 3	Factor 4	Factor 5	Factor 6	Factor 7	Factor 8
Item 11	**0.50**	0.28	0.29	0.36	0.43	0.03	−0.12	0.15
Item 12	**0.55**	0.39	0.16	0.41	0.30	0.10	− 0.14	0.18
Item 13	**0.60**	0.31	0.27	0.40	0.50	0.19	−0.02	0.28
Item 15	**0.62**	0.40	0.19	0.38	0.36	0.12	−0.07	0.23
Item 17	**0.57**	0.31	0.22	0.37	0.37	0.07	−0.13	0.16
Item 18	**0.69**	0.41	0.21	0.44	0.36	0.02	−0.08	0.30
Item 25	**0.47**	0.33	0.09	0.17	0.16	0.19	−0.11	0.18
Item 30	**0.48**	0.25	0.23	0.25	0.47	0.09	−0.09	0.24
Item 32	**0.57**	0.48	0.16	0.34	0.37	0.13	−0.10	0.28
Item 23	***0.49***	**0.43**	0.18	0.32	0.28	0.17	−0.03	0.11
Item 27	0.37	**0.57**	0.10	0.21	0.22	0.07	−0.05	0.13
Item 28	0.41	**0.60**	0.09	0.21	0.25	0.20	−0.03	0.15
Item 29	0.38	**0.49**	0.00	0.16	0.22	0.14	−0.14	0.13
Item 1	0.27	0.11	**0.46**	0.23	0.43	0.01	−0.01	0.17
Item 2	0.24	0.11	**0.52**	0.39	0.26	−0.03	0.01	0.27
Item 3	0.16	−0.01	**0.39**	0.13	0.20	−0.11	0.03	0.17
Item 6	0.20	0.08	**0.38**	0.21	0.25	0.10	−0.07	0.22
Item 20	0.35	0.14	0.26	**0.47**	0.24	−0.04	−0.01	0.19
Item 21	***0.50***	0.32	0.12	**0.49**	0.27	0.01	−0.02	0.18
Item 22	0.55	0.37	0.32	**0.58**	0.35	0.05	0.03	0.23
Item 31	0.54	0.36	0.25	0.32	**0.57**	0.18	−0.06	0.34
Item 39	0.22	0.12	0.16	0.23	**0.26**	−0.04	0.05	0.06
Item 4	***0.45***	0.28	***0.35***	0.14	**0.30**	0.15	−0.10	0.19
Item 40	0.29	0.15	0.22	0.27	**0.37**	0.10	−0.02	0.08
Item 5	0.35	0.18	0.34	0.08	**0.45**	0.10	0.03	0.23
Item 16	***0.21***	***0.24***	0.11	0.06	0.16	**0.19**	0.03	0.13
Item 36	0.17	0.08	−0.05	0.03	0.07	**0.32**	0.05	0.13
Item 37	0.00	−0.04	−0.06	0.03	−0.03	**0.27**	−0.04	0.01
Item 38	0.05	0.20	−0.13	−0.05	0.14	**0.27**	−0.07	0.05
Item 35	−0.16	−0.13	− 0.08	0.00	− 0.01	−0.03	**0.39**	0.07
Item 7	−0.10	−0.08	0.03	0.03	−0.02	0.01	**0.39**	−0.02
Item 10	0.13	0.00	***0.31***	0.20	0.23	−0.04	0.08	**0.01**
Item 19	***0.35***	0.31	0.06	0.22	0.16	0.19	0.01	**0.10**
Item 8	0.20	0.07	0.28	0.09	***0.31***	0.07	0.11	**0.23**
Item 9	0.09	0.06	−0.05	−0.01	−0.05	0.03	− 0.06	**−0.14**

Inter-item correlations within factors (in bold) close to 0.5 except for factor 6 and 8. Between-subscale inter-item correlations were lower except for items 4, 16, 21,23 (in italic)

**Table 6:** Inter-subscale correlation matrix a coefficient of under 0.4 confirms the independence of dimensions

	Factor 1	Factor 2	Factor 3	Factor 4	Factor 5	Factor 6	Factor 7
Factor 2	0.53						
Factor 3	0.30	0.09					
Factor 4	0.52	0.27	0.32				
Factor 5	0.56	0.31	0.40	0.31			
Factor 6	0.14	0.19	−0.02	0.01	0.11		
Factor 7	−0.15	− 0.11	− 0.02	0.01	− 0.02	−0.01	
Factor 8	0.32	0.18	0.29	0.24	0.29	0.11	0.06

#### Reproducibility

It was not possible to test reproducibility as only 10 patients were able to perform the test/retest procedure, due to the unstable health of this group of terminally ill patients.

#### Stability

We confirmed stability using the Promax and Oblimin rotation in PCA with similar results (not presented) of those of the Varimax rotation (Table 3). The factorial structure was found globally maintained across the different sub-groups: the proportion of identically attributed items were respectively for .46 for age (permutation test *p* = .92), .37 for sex (*p* = .52), .43 for PPS ≤ 30% (*p* = .84) and .37 for PPS ≥ 60% (p = .52), .4 for settings (*p* = .7). Among most stable items were those related to availability of caregivers, information, pain and serenity. Then, the mean scores of factors were close across sub-groups, except for factor 1 (availability) and 2 (serenity) which were significantly better in the palliative care unit, but this statistical difference between such high scores may not be clinically relevant (Table 7).

**Table 7:** Comparison of the means of the scores (sd) according to age, sex, PPS score, and care structure

		Factor 1	Factor 2	Factor 3	Factor 4	Factor 5	Factor 6	Factor 7	Factor 8
Male (*n* = 78)	Mean (std)	3.69 (0.45)	3.43 (0.68)	3.40 (0.72)	3.72 (0.63)	3.45 (0.62)	3.75 (0.47)	3.79 (0.61)	3.64 (0.36)
Female (*n* = 114)	Mean (std)	3.68 (0.43)	3.51 (0.62)	3.32 (0.66)	3.74 (0.53)	3.47 (0.57)	3.83 (0.34)	3.79 (0.58)	3.67 (0.39)
	p	0.81	0.38	0.19	0.78	0.88	0.72	0.85	0.3
Outside USP (*n* = 112)	Mean (std)	3.60 (0.47)	3.23 (0.69)	3.42 (0.67)	3.71 (0.61)	3.42 (0.59)	3.75 (0.45)	3.85 (0.49)	3.64 (0.39)
USP (*n* = 80)	Mean (std)	3.80 (0.35)	3.83 (0.36)	3.26 (0.69)	3.75 (0.52)	3.52 (0.58)	3.86 (0.32)	3.72 (0.71)	3.68 (0.35)
	p	< 0.0001	< 0.0001	0.06	0.96	0.08	0.06	0.17	0.63
PPS ≥ 60% (*n* = 41)	Mean (std)	3.65 (0.47)	3.31 (0.78)	3.39 (0.65)	3.82 (0.60)	3.54 (0.50)	3.78 (0.41)	3.86 (0.39)	3.72 (0.32)
40–50% (*n* = 89)	Mean (std)	3.71 (0.43)	3.50 (0.62)	3.35 (0.69)	3.71 (0.53)	3.44 (0.64)	3.80 (0.42)	3.79 (0.61)	3.67 (0.40)
≤ 30% (*n* = 60)	Mean (std)	3.67 (0.43)	3.54 (0.59)	3.35 (0.70)	3.69 (0.61)	3.45 (0.54)	3.80 (0.38)	3.74 (0.67)	3.59 (0.38)
	p	0.76	0.35	0.99	0.13	0.74	0.96	0.73	0.1
< 65 yrs (*n* = 73)	Mean (std)	3.72 (0.42)	3.51 (0.65)	3.50 (0.61)	3.88 (0.36)	3.48 (0.58)	3.70 (0.48)	3.81 (0.62)	3.67 (0.38)
≥ 65 yrs (*n* = 118)	Mean (std)	3.66 (0.44)	3.47 (0.65)	3.26 (0.72)	3.64 (0.65)	3.45 (0.6)	3.85 (0.33)	3.78 (0.58)	3.65 (0.37)
	p	0.23	0.61	0.02	0.01	0.75	0.01	0.42	0.67
Partially satisfied (n = 6)	Mean (std)	2.75 (0.40)	2.75 (1)	2.6 (0.98)	2.89 (0.81)	2.4 (0.79)	3.57 (0.4)	4 (0)	3.13 (0.54)
Satisfied (*n* = 87)	Mean (std)	3.5 (0.49)	3.24 (0.71)	3.18 (0.72)	3.62 (0.7)	3.31 (0.64)	3.75 (0.45)	3.81 (0.56)	3.6 (0.39)
Very satisfied (*n* = 97)	Mean (std)	3.9 (0.15)	3.75 (0.4)	3.55 (0.55)	3.87 (0.3)	3.65 (0.39)	3.86 (0.34)	3.78 (0.61)	3.74 (0.32)
	p	< 0.0001	< 0.0001	< 0.0001	< 0.0001	< 0.0001	0.02	0.62	0.001

#### External validation

Global satisfaction with care was inversely correlated with the global symptoms score of the MDASI and the interference of symptoms on the quality of life score (Table 8). Each factor of the questionnaire correlated with the global satisfaction with care, except factor 7. Factor 1 (availability) was clearly negatively correlated with both the symptoms score and the interference with quality of life score.

**Table 8:** Correlation between global satisfaction and the symptoms score (MDASI) and interference of the symptoms score with each factor of the Quali-Palli-Pat

	Spearman coefficient	*p*
Symptoms score	−0.22	0.003
Impact score	−0.25	0.0007
Factor 1	0.56	< 0.0001
Factor 2	0.42	< 0.0001
Factor 3	0.32	< 0.0001
Factor 4	0.27	0.0001
Factor 5	0.34	< 0.0001
Factor 6	0.18	0.01
Factor 7	−0.03	NS
Factor 8	0.25	0.0001
Correlation with the symptom score of the MDASI		
Factor 1	−0.25	0.0006
Factor 2	−0.1	0.16
Factor 4	−0.18	0.02
Correlation with the interference of symptoms on the quality of life score		
Factor 1	−0.26	0.0005
Factor 2	−0.22	0.003
Factor 4	−0.1	0.17

## Discussion

### Quali-Palli-pat: A validated tool to explore eight domains

In this multicenter prospective study, we created and validated a French questionnaire – Quali-Palli-Pat – that evaluates the quality of palliative care and the satisfaction with care from the patients’ perspective. We started with an initial questionnaire of 43 items. After analysis, the final questionnaire includes 35 items that explore the eight following domains: availability of caregivers, serenity, quality of information, pain management, willingness to listen to patients, psychosocial and spiritual aspects, right of refusal, and respect of the patients. The questionnaire concludes with a 36th question concerning general satisfaction. These eight dimensions, obtained after PCA, are in accordance with the six initially expected, but also include the theme of pain management and separate items which initially belonged to the relationship with doctors and caregivers. These results highlight major themes previously described in the palliative care literature and finally correspond to patient priorities, such as the availability of caregivers or the need for serenity [3, 14, 39–42]. By exploring these eight dimensions, Quali-Palli-Pat differs from unidimensional satisfaction questionnaires, such as Famcare Pat [19, 43]. The stability of these domains was maintained across different sub-groups of patients (sex, age, PPS) and healthcare settings.

### External validation and relation with satisfaction and quality of life

Our results indicate that global satisfaction is linked primarily to the global score of the MDASI symptom assessment scale and secondly to the interference of symptoms on the quality of life score. An important factor, such as the availability of caregivers, was linked both to the symptom score and interference score. However, Quali-Palli-Pat is neither a questionnaire of symptom assessment nor of quality of life. Satisfaction with care and quality of care are not interchangeable measures [44]. Our results indicate that all but one (right to refuse) of the explored dimensions positively correlated with the score of general satisfaction and probably directly contributed to it, as previously suggested [39, 41, 44, 45]. As quality of care may be some externally assessed measure, satisfaction with care deals not only with quality from a technical perspective but also with discrepancies between patients’ attempts and provision of care. [45]. Health status, quality of care, satisfaction with care, and quality of life appear to be in a dynamic loop [17]. We observed that a relative well-being (MDASI score) is associated to the satisfaction, related to obvious efforts of the caregivers mediated through interpersonal communication, as postulated by Stewart et al. [45]. However, according to the integrative response shift model proposed by Sprangers and Schwartz, a worsening of health status may modify the tolerance of the patient, and thus paradoxically maintain the level of the quality of life [46]. A similar process may have affected our measures of quality of care and satisfaction, found to be stable irrespective of the PPS, such as the recently reported independence between quality of life and ECOG performance status [37].

### Strengths of the method

The strength of the method used to create and validate Quali-Palli-Pat makes it a theoretically sound and valid instrument, based on scoring of measurement properties as formalized in the COSMIN checklist (www.cosmin.nl/), which includes internal consistency, reliability, measurement error, content validity, construct validity, criterion validity, and responsiveness [47, 48]. Quali-Palli-Pat meets most of these criteria. Except for reliability, measurement error, and responsiveness whose evaluation was hampered by the rapid deterioration of patients, all other criteria were excellent or good. Sample size and the methods appeared to be good both for dimensionality and internal consistency analysis, while also managing missing data. Concerning sample size, we had an initial sample-to-variable ratio of 5:1 and final variable-to-factor ratio of 4:1. Our sample size thus appeared to be adequate, considering the mean variable-to factor ratio and the relatively high value of the loadings (mean = 0.55) [49]. Content validity was built on a qualitative study among patients, and was confirmed by the easy interpretability of the final subscales. Construct validity was based on exploratory factor analysis and we verified the stability of the structure. Criterion validity mainly confirmed a priori hypotheses, facing global satisfaction or relation with the impact of the disease. Finally, the questionnaire was administered in various settings, to patients of various ages and physical status, thus enhancing the generalization of the study findings, although future validation should include home hospitalized patients.

### Quali-Palli-pat: A useful tool for public health policy makers

We conceived Quali-Palli-patient as a diagnostic tool that provides a quality profile at the service level, highlighting domains which need improvement, rather than a unique and absolute score, which is difficult to interpret. Evaluation at the patient level is not appropriate, as evaluating and comparing individuals within a setting requires very high internal consistency, high precision and adjustment on patient status while there is the problem of the lack of independence of the patient from the caregiver. Our instrument may be used as a pre- and post-measurement tool in quality improvement interventions, as recommended by the e-satis program of the French National Authority for Health with periodic audits of satisfaction of hospitalized patients [50]. Periodically using this tool to compare various settings of palliative care could provide the means to better adapt public health policies and funding.

### Communication between patients and health providers

Quali-Palli-Pat largely explores the communication between patients and health providers. This is an extremely important domain, especially when deciding to stop specific treatments, such as chemotherapy, and at the beginning of exclusive palliative care. Teno et al. previously highlighted this domain and developed a specific tool focusing on information needs, especially at the time of diagnosis or making decisions concerning treatment [41]. In our study, this domain showed the lowest values, highlighting the necessity of further improvement, especially in palliative care units. A single item concerned spirituality (“I can talk to someone about philosophical or religious issues if I wish”) but with a rate of 27% of NA, despite its harmless formulation, this item would have been deleted according to our rules. However, it was retained by the working group because spirituality is an essential component of palliative care [51, 52]. The NA response can be explained by the specificity of French secularism, which considers religious or spiritual domains as private; French patients cannot understand the relationship between care, quality of care, and spirituality [53].

### Limits of the study

Our study had limitations. First, we used a sample of inpatients hospitalized in palliative care units or academic hospitals involved in quality of palliative care. This choice was probably not representative of the general population of palliative care inpatients, nor of the numerous settings tagged with palliative care services, ours being volunteers. This could explain the ceiling effect frequently observed and justifies retaining some items, despite this effect. The choice of retaining some items, despite their ceiling effect, should be confirmed in a future confirmatory study including a more diverse population of structures and patients.

Second, reproducibility could not be assessed due to the rapid evolution of the health status of the patients. Testing reliability requires a test/retest in which patients remain stable for a reasonable period, perhaps a week [1]. This short timeframe is far too long for hospitalized palliative care patients, because the median overall survival in palliative care units is approximately 12 days, during which patients quickly and inevitably get worse. Thus, it was not possible to test reliability and sensitivity to change, as in other studies [1]. Quali-Palli-Pat, like all instruments of this kind, can only be used by fully communicative patients, which does not reflect the palliative care population, especially at the end of life [54]. Thus, we plan to create and validate the Quali-Palli-Fam questionnaire, intended for relatives. We did not estimate the relative weight of each of the eight domains, as tested in other settings [42]*.* We believe that it would be too difficult for terminally ill tired patients to disentangle the two related questions of perceived quality and subjective importance, item by item [43]. This could eventually be done in a subsequent confirmatory survey, which could also verify the existence of these eight domains in other contexts.

Although the questionnaire was created to be as brief, understandable, and easy as possible, 80% of patients required assistance to complete it. Response bias was reduced by the fact that the interviewer was a clinical research associate, independent from the caregivers of the hospitals [55]. This raises the question of who will assist in the completion of the questionnaire for periodic assessments. In our recent experience, it is possible to involve volunteers. However, some patients with delirium or vigilance disorders will never complete it, explaining the lack of patients with brain tumors in our data. The Quali-Palli-Fam, which explores the family perspective, should at least partially compensate for this.

## Conclusion

Quali-Palli-Pat appears a valid, reliable, and well-accepted French tool that explores the quality of palliative care and the satisfaction in patients with advanced disease, hospitalized in various settings, and is ready for use in the French language (Additional file 1: French Quali-Palli-Pat). Providing a voice for the most vulnerable patients is the right way to assess the gap between their needs and provided care. This questionnaire reflects the quality of a structure and is not considered at the individual level. Periodic patient audits would help care teams to improve the quality of palliative care. It must be complemented in the future by a relatives’ questionnaire.

## Additional file

Additional file 1: Quali-Palli-Pat : Questionnaire sur la qualité de la prise en charge. Original French version of the questionnaire concerning patients’ perspectives of the quality of palliative care: Quali-Palli-Pat. (DOCX 44 kb)

## Abbreviations

EFA: Exploratory factor analysis
KMO: Kaiser–Meyer–Olkin
MD: Missing data
MDASI: MD Anderson Symptom Inventory
NA: Not applicable
NR: Non response
PCA: Principal component analysis
PPS: Palliative performance scale
