# Supplementary material for: Development and validation of a French questionnaire concerning patients’ perspectives of the quality of palliative care: the QUALI-PALLI-Patient
Source: BMC Palliat Care. 2019 Feb 11;18:19. doi: 10.1186/s12904-019-0403-z (PMC6369559; doi:10.1186/s12904-019-0403-z)
Supplement: Supplementary file 1 — Quali-Palli-Pat : Questionnaire sur la qualité de la prise en charge. Original French version of the questionnaire concerning patients’ perspectives of the quality of palliative care: Quali-Palli-Pat. (DOCX 44 kb) [file 12904_2019_403_MOESM1_ESM.docx]

**Quali-Palli-Pat : Questionnaire sur la qualité de la prise en charge**

**N° Chambre** :

**Remplissage du questionnaire** : Seul Avec aide

**Date** : **Age** : **Sexe** : Féminin Masculin

**Donnez-nous vos impressions pour améliorer la qualité de nos soins**

L’établissement a besoin de connaître votre vécu pour améliorer les soins et l’accompagnement des personnes hospitalisées et de leurs proches. N’hésitez pas à nous faire savoir ce qui va bien ainsi que les difficultés rencontrées : tout a son importance. Merci de prendre le temps de remplir ce questionnaire.

Choisissez la réponse la plus proche de ce que vous pensez. Cochez une réponse par ligne.

**NC** : Non concerné

**Ma participation :**

Je réponds Je ne veux pas répondre  Je ne peux pas répondre

| **Disponibilité des soignants pour répondre à mes besoins** | | | | | |
| --- | --- | --- | --- | --- | --- |
|  | **Pas du tout** | **Pas vraiment** | **Presque** | **Tout à fait** | **NC** |
| Je vois un soignant aussi souvent que je le souhaite ou que j’en ai besoin | O | O | O | O | O |
| Les soignants répondent rapidement à mes appels | O | O | O | O | O |
| On fait le maximum si je suis angoissé(e), inquiet(e) ou triste | O | O | O | O | O |
| Pour mes gestes quotidiens, on fait le maximum pour m’aider | O | O | O | O | O |
| Les soignants sont disponibles pour m’écouter | O | O | O | O | O |
| Les soignants montrent une réelle disponibilité et attention à chaque fois qu’ils entrent dans la chambre | O | O | O | O | O |
| Pour me laver, j’obtiens une aide très adaptée | O | O | O | O | O |
| Les infirmièr(e)s font tout pour se rendre disponibles pour moi | O | O | O | O | O |
| Je ressens une bonne coordination dans le service | O | O | O | O | O |

| **Attention à ma sérénité : douceur, repos, environnement, respect de mes rythmes** | | | | | |
| --- | --- | --- | --- | --- | --- |
|  | **Pas du tout** | **Pas vraiment** | **Presque** | **Tout à fait** | **NC** |
| Les soignants font preuve de douceur dans les gestes de soins | O | O | O | O | O |
| La chambre et le service constituent un environnement calme et serein | O | O | O | O | O |
| Je peux me reposer autant que je le souhaite | O | O | O | O | O |
| Les soignants respectent mon rythme de repos, par exemple mon heure de réveil | O | O | O | O | O |

| **Qualité de l’information (santé, soins, traitement) et mon implication dans les décisions** | | | | | |
| --- | --- | --- | --- | --- | --- |
|  | **Pas du tout** | **Pas vraiment** | **Presque** | **Tout à fait** | **NC** |
| **J’ai obtenu des informations très claires sur** : |  |  |  |  |  |
| - L’évolution de mon état de santé | O | O | O | O | O |
| - L’objectif des traitements (médicament, opération) | O | O | O | O | O |
| - Les effets indésirables possibles des traitements | O | O | O | O | O |
| Je suis impliqué(e) dans les soins et les décisions me concernant | O | O | O | O | O |

| **Prise en charge de la douleur** | | | | | |
| --- | --- | --- | --- | --- | --- |
|  | **Pas du tout** | **Pas vraiment** | **Presque** | **Tout à fait** | **NC** |
| Je suis régulièrement interrogé(e) sur ma douleur | O | O | O | O | O |
| Je suis rapidement pris en charge pour ma douleur quand je la signale | O | O | O | O | O |
| Je bénéficie d’une prise en charge de la douleur avant chaque toilette ou soin si nécessaire | O | O | O | O | O |

| **Qualité d’écoute des soignants** | | | | | |
| --- | --- | --- | --- | --- | --- |
|  | **Pas du tout** | **Pas vraiment** | **Presque** | **Tout à fait** | **NC** |
| J’obtiens facilement des informations quel que soit mon interlocuteur | O | O | O | O | O |
| Les médecins répondent à toutes mes questions | O | O | O | O | O |
| Les médecins font tout pour se rendre disponibles pour moi | O | O | O | O | O |
| Les médecins me demandent mon autorisation avant d’informer mes proches sur mon état de santé | O | O | O | O | O |
| Mes proches obtiennent des informations claires et compréhensibles sur mon état de santé | O | O | O | O | O |

| **Dimensions psychologique, sociale et spirituelle** | | | | | |
| --- | --- | --- | --- | --- | --- |
|  | **Pas du tout** | **Pas vraiment** | **Presque** | **Tout à fait** | **NC** |
| J’ai été perturbé(e) par les propos tenus par les médecins pendant la visite | O | O | O | O | O |
| Je peux bénéficier d’un entretien avec un psychologue au moment où j’en ai besoin | O | O | O | O | O |
| Je peux bénéficier d’un entretien avec une assistante sociale au moment où j’en ai besoin | O | O | O | O | O |
| Je peux bénéficier d’un entretien à caractère philosophique ou religieux si je le désire | O | O | O | O | O |

| **Possibilité de refus de certains soins ou de la présence de bénévoles** | | | | | |
| --- | --- | --- | --- | --- | --- |
|  | **Pas du tout** | **Pas vraiment** | **Presque** | **Tout à fait** | **NC** |
| Je peux éventuellement refuser certains soins | O | O | O | O | O |
| J’ai pu refuser la présence de bénévoles | O | O | O | O | O |

| **Respect du patient en tant qu’acteur de sa prise en charge** | | | | | |
| --- | --- | --- | --- | --- | --- |
|  | **Pas du tout** | **Pas vraiment** | **Presque** | **Tout à fait** | **NC** |
| Les médecins m’écoutent et prennent en compte ce que je dis | O | O | O | O | O |
| Certains soins ou interventions sont réalisés sans mon accord | O | O | O | O | O |
| Je connais le(s) médecin(s) qui s’occupe(nt) de moi dans le service | O | O | O | O | O |
| Il arrive aux soignants de parler entre eux comme si je n’étais pas là | O | O | O | O | O |

| **Satisfaction globale** | | | | |
| --- | --- | --- | --- | --- |
|  | Pas satisfait du tout | Peu satisfait | Satisfait | Très satisfait |
| Au final, quel est votre niveau de satisfaction ? | O | O | O | O |

| **A votre avis, que faudrait-il améliorer en priorité dans ce service ?** |
| --- |
|  |
|  |
|  |
|  |
|  |
|  |
|  |
|  |
|  |
